# Exploring the Influence of Biochar-Supported Nano-Iron Oxide on Phosphorus Speciation Transformation and Bacterial Community Structure in Aerobic Pig Manure Composting Processes

**DOI:** 10.3390/microorganisms12122593

**Published:** 2024-12-14

**Authors:** Ning Yuan, Kang Wang, Mengyue Liang, Jia Zhou, Rui Yu

**Affiliations:** 1 College of Geographic Science, Harbin Normal University, Harbin 150025, China; yuanning@iga.ac.cn (N.Y.); kangwang2021@outlook.com (K.W.); liangmengyue@iga.ac.cn (M.L.); 2 State Key Laboratory of Black Soils Conservation and Utilization, Northeast Institute of Geography and Agroecology, Chinese Academy of Sciences, Changchun 130102, China

**Keywords:** BC-Fe_3_O_4_NPs, aerobic composting, phosphorus speciation, metagenomics

## Abstract

Existing studies have demonstrated the positive effects of nano-sized iron oxide on compost maturity, yet the impact of nano-sized iron oxide on phosphorus speciation and bacterial communities during the composting process remains unclear. In this study, pig manure and straw were used as raw materials, with biochar-supported nano-sized iron oxide (BC-Fe_3_O_4_NPs) as an additive and calcium peroxide (CaO_2_) as a co-agent, to conduct an aerobic composting experiment with pig manure. Four treatments were tested: CK (control), F1 (1% BC-Fe_3_O_4_NPs), F2 (5% BC-Fe_3_O_4_NPs), and F3 (5% BC-Fe_3_O_4_NPs + 5% CaO_2_). Key findings include the following. (1) BC-Fe_3_O_4_NPs increased compost temperatures, with F3 reaching 61℃; F1 showed optimal maturity (C/N ratio: 12.90). (2) BC-Fe_3_O_4_NPs promoted stable phosphorus forms; Residual-P proportions were higher in F1, F2, and F3 (25.81%, 51.16%, 51.68%) than CK (19.32%). (3) Bacterial phyla *Firmicutes*, *Actinobacteria,* and *Proteobacteria* dominated. BC-Fe_3_O_4_NPs altered community composition, especially on day 7. *Firmicutes* dominated CK, F1, and F3; *Proteobacteria* dominated F2. At the genus level, day 7 showed *Corynebacterium* (CK), *Clostridum* (F1, F3), and *Caldibacillus* (F2) as predominant. (4) Pearson correlation analysis revealed shifted correlations between phosphorus forms and bacterial phyla after BC-Fe_3_O_4_NPs addition. *Firmicutes* positively correlated with NaOH-OP in F1 during the thermophilic phase, facilitating phosphate release and adsorption by BC-Fe_3_O_4_NPs. The significance of correlations diminished with increasing additive concentration; in F3, all phyla positively correlated with various phosphorus forms.

## 1. Introduction

Phosphorus (P), although ubiquitous in nature, remains one of the most challenging nutrients for plant uptake [1]. In China, the predominant approach to replenishing soil P involves the application of chemical P fertilizers. Nonetheless, the raw material for these fertilizers, phosphate rock, is a finite resource, and its excessive extraction has disrupted the natural P cycle [2]. Additionally, the overuse of P fertilizers has led to the accumulation of P in agricultural soils, causing environmental issues, such as water eutrophication and non-point source pollution [3,4]. Animal manure, which is abundant in P and offers a mix of easily mineralizable and organically bound P [5], presents a promising alternative to chemical P fertilizers.

Aerobic composting represents one of the most crucial technologies for the resource recovery of animal manure [6], effectively sanitizing pathogens and weed seeds contained within the manure via high-temperature conditions [7]. Elucidating the transformations of P species during composting is essential to mitigating environmental issues stemming from soil P surplus induced by organic fertilizers and to facilitate site-specific P fertilizer application, thereby preventing wastage associated with uniform application strategies [8]. Present research efforts commonly involve the incorporation of additives during composting to modulate P speciation conversions [9]. In a study by Liu [10], glucose, biochar, and wood peat were utilized as additives in the aerobic composting of chicken manure. The findings revealed that the wood-peat-amended group exhibited the highest NaOH-OP fraction at 8.7%, while the glucose-amended group had the highest HCl-IP fraction at 35.2%. Zhang’s [11] research indicated that incorporating 5% yellow phosphorus residue into pig manure and straw aerobic composting led to a predominance of HCl-IP in the final compost product. Similarly, Zhang found that adding 5% yellow phosphorus residue to pig manure and straw aerobic compost resulted in HCl-IP being the dominant phosphorus form. Wei’s [12] study concluded that the addition of biochar to cattle manure and straw aerobic compost could restrict phosphorus effectiveness.

Nanomaterials, characterized by their large specific surface area and small particle size, have been widely utilized in composting experiments [13,14,15]. Fe_3_O_4_NPs serve as advanced redox catalysts, providing both Fe^2+^ and Fe^3+^ ions. Fe^2+^ can be oxidized to Fe^3+^ by H_2_O_2_, generating hydroxyl radicals (·OH). These ·OH radicals are capable of disrupting the lignocellulosic structure, thereby promoting the maturity of the compost [16]. Studies have shown that Fe_3_O_4_NPs can significantly enhance the composting process [15,17]. However, there is a paucity of research on the impact of iron-based oxidation additives on phosphorus speciation in aerobic composting.

Magnetic nanoparticles are inherently prone to aggregation, a phenomenon that can markedly diminish their catalytic degradation efficacy in practical applications. To address this issue, our study opted to immobilize Fe_3_O_4_ nanoparticles onto biochar. Biochar, with its distinctive porous structure, not only facilitates enhanced oxygen exchange but also offers the added benefit of being cost-effective [18]. CaO_2_, as a green and safe oxygen-releasing agent, slowly decomposes upon contact with water to release oxygen [19]. The incorporation of CaO_2_ into composting experiments can more effectively supply H_2_O_2_, accelerating the conversion of Fe^2+^ to Fe^3+^, and provide oxygen to prevent anaerobic conditions within the compost pile.

Furthermore, biochar and Fe_3_O_4_NPs can offer a conducive environment for the proliferation, growth, and development of microorganisms, thereby modulating the diversity of microbial communities [12]. During the composting process, microorganisms not only dictate the rate of organic matter degradation [20] but also directly participate in the transformation of phosphorus and the regulation of its bioavailability [21]. This underscores the complexity of phosphorus speciation transformation within the composting context. Nonetheless, there remains a dearth of investigation into the impact of BC-Fe_3_O_4_NPs on bacterial communities.

Building on this foundation, the present study conducted a 50-day aerobic composting experiment using pig manure and straw in 100 L PVC containers, with the addition of varying concentrations of BC-Fe_3_O_4_NPs and CaO_2_. The Hedley phosphorus fractionation method and metagenomic sequencing were employed to analyze the transformations of phosphorus species and the diversity and succession of bacterial communities during the composting process. We hypothesize the following. (1) BC-Fe_3_O_4_NPs and CaO_2_ will influence the transformation of phosphorus species towards more stable forms. (2) The addition of BC-Fe_3_O_4_NPs and CaO_2_ will induce significant differences in bacterial communities during the composting process. (3) BC-Fe_3_O_4_NPs and CaO_2_ will impact the relationship between dominant microbial populations and phosphorus species.

## 2. Materials and Methods

### 2.1. Biochar Loading Experiment

For the preparation of this study, Fe_3_O_4_NPs with a diameter of 20 nm, exhibiting a spherical particulate morphology, were procured from Shanghai Macklin Biochemical Technology Co., Ltd. (Shanghai, China). Rice-derived biochar was obtained from Nanjing Qinfeng Straw Technology Co., Ltd., located in Nanjing, China. CaO_2_ with a purity of 75% was purchased from Shaoxing Shangyu Jiehua Co., Ltd. (Shaoxing, China). The synthesis procedure for BC-Fe_3_O_4_NPs is as follows. Biochar and Fe_3_O_4_NPs were precisely weighed according to a predetermined ratio and placed into a beaker. The mixture was supplemented with 100 mL of a highly purified grade of anhydrous ethanol (Sinopharm Chemical Reagent Co., Ltd., Shanghai, China) and stirred thoroughly. Subsequently, the mixture underwent ultrasonication for 1 h to ensure thorough dispersion. After this treatment, the mixture was dried in an oven, followed by grinding to achieve homogeneity. The resultant powder was then calcined in a muffle furnace for 2 h. Upon completion of the calcination process, the material was again ground and subsequently stored in sealed containers for future use. The BC-Fe_3_O_4_NPs samples were submitted to Hongrui Research Service Centre, Shanghai, for Brunauer–Emmett–Teller (BET) surface area analysis (Autosorb-iQ, Quantachrome, East Lyme, CT, USA) and scanning electron microscopy with energy dispersive X-ray spectroscopy (SEM-EDS) examination (Gemini 300, ZEISS, Oberkochen, Germany). The total carbon (TC) and total nitrogen (TN) contents of BC-Fe_3_O_4_NPs were determined using a carbon–nitrogen element analyzer (Vario Macro cube, Langenselbold, Germany). A specified amount of BC-Fe_3_O_4_NPs was mixed with ultrapure water at a ratio of 1:10, oscillated for 16 h, and then centrifuged at 5000 rpm for 10 min to obtain the supernatant. The supernatant was subsequently filtered through filter paper, and the filtered liquid was used to measure its pH (PHS-3C, Leici, Shanghai, China) and electrical conductivity (EC) (DDS-11A, Leici, Shanghai, China).

### 2.2. Experimental Composting Process

The raw materials for composting comprised fresh pig manure and maize straws from large-scale and family farms in Gongzhuling City, Jilin Province, China. The corn stalks were crushed to a length of 2–3 cm and then uniformly mixed with pig manure in a ratio of 3:1, and the moisture content was adjusted to 65%. The moisture content of pig manure and straw was determined using the constant-weight drying method. Firstly, a clean aluminum box was weighed, and its weight was recorded as *N*1. A certain amount of fresh pig manure or straw was then placed into the aluminum box and weighed again to obtain the total weight, *N*2. Subsequently, the sample was placed in an electric hot air drying oven at 105 °C and dried to a constant weight. After cooling, the net weight of the sample was measured and recorded as *N*3. The moisture content was calculated according to Formula (1).


(1) $$\text{ Moisture content }\;(\%)=\frac{N2-N3}{N2-N1}\times 100\%$$

A portion of the pig manure and straw was air-dried, crushed, and sieved through a 100-mesh screen. The TC and TN contents were then determined using a carbon–nitrogen element analyzer (Vario Macro cube, Langenselbold, Germany). Another portion of the pig manure and straw was freeze-dried, crushed, and also sieved through a 100-mesh screen. The freeze-dried samples were mixed with ultrapure water at a ratio of 1:10, oscillated for 1 h, and then centrifuged at 5000 rpm for 10 min to obtain the supernatant. The pH and EC of the supernatant were measured using a pH meter (PHS-3C, Leici, Shanghai, China) and an electrical conductivity meter (DDS-11A, Leici, Shanghai, China), respectively. The physicochemical properties of the compost raw materials are presented in Table S1 of the Supplementary Materials.

The treatments with 0%, 1%, and 5% of BC-Fe_3_O_4_NPs and 5% of BC-Fe_3_O_4_NPs+CaO_2_ were denoted as the control group (CK), F1, F2, and F3, respectively. The composting experiment was conducted in a 100 L PVC bucket for 50 d. The fundamental characteristics of pig manure and maize straw are presented in Table S1. The schematic diagram of the device is shown in Figure S1.

Temperature readings were recorded daily at 10:00 AM and 3:00 PM to monitor the composting process. During the composting period, manual turning and aeration were conducted on days 2, 4, 7, 14, 21, 28, 35, and 50 using a drum mixer to ensure uniform mixing of the materials. Following mixing, random samples were collected for analysis.

One portion of each sample was air-dried, subsequently shattered, and sieved through a 100-mesh screen for the determination of total carbon (TC) and total nitrogen (TN). Another portion was freeze-dried, shattered, and also sieved through a 100-mesh screen for the measurement of pH, electrical conductivity (EC), and P speciation. An additional sample was stored at −80 °C and sent to Nanjing Personal Biotechnology Co., Ltd. for metagenomic sequencing.

Furthermore, specific samples were collected on days 0, 7, 28, and 50, representing the key stages of composting: the heating–high-temperature phase, the high temperature–cooling phase, the cooling–maturation phase, and the full maturation phase, respectively. These samples were used to analyze the transformation of P speciation during different composting stages.

### 2.3. Physical and Chemical Properties of the Composting

An appropriate quantity of the sample was selected and placed in an electric heating blast drying oven at 105 ℃ until a constant mass was obtained. After 24 h, the sample was removed from the oven and weighed. The moisture content was then calculated. The lyophilized sample was mixed with ultrapure water in a 1:10 ratio, and the supernatant was centrifuged at 5000 rpm for 10 min after shaking for 1 h. A pH meter (PHS-3C, Leici, Shanghai, China) and a conductivity meter (DDS-11A, Leici, Shanghai, China) were used to determine the pH and EC. Total carbon and nitrogen contents were measured using a carbon and nitrogen analyzer (Vario Macro cube, Langenselbold, Germany). We placed 10 plump Chinese cabbage seeds into a Petri dish, added 5 mL of the aforementioned supernatant, and incubated them in darkness at 25 °C for 48 h. Then, we measured the root length and calculated the seed germination index (*GI*) using Formula (2).


(2) $$\text{GI }=\frac{\text{Sample }\;\text{Germination }\;\text{Rate }\times \text{Sample }\;\text{Average }\;\text{Root }\;\text{Length }}{\text{Blank }\;\text{Germination }\;\text{Rate }\times \text{Blank }\;\text{Average }\;\text{Root }\;\text{Length }}\times 100\%$$

### 2.4. P Forms

The concentrations of total P (TP) of compost samples were tested with the molybdate-blue colorimetric method after it was digested with HNO_3_-HClO_4_ [12]. The P forms were evaluated using a modified version of Hedley’s method [22]. Specifically, a 0.3000 g sample was taken. The H_2_O-IP (inorganic phosphorus extracted with water), NaHCO_3_-IP (inorganic phosphorus extracted with NaHCO_3_), NaOH-IP (inorganic phosphorus extracted with NaOH), and HCl-IP (inorganic phosphorus extracted with HCl) morphologies were obtained by adding ultrapure water, 0.50 mol·L^−1^ NaHCO_3_, 0.10 mol·L^−1^ NaOH, and 1.00 mol·L^−1^ HCl in a 1:10 ratio. The residue was digested and extracted with a H_2_SO_4_-H_2_O_2_ solution. The contents of the aforementioned P forms were determined using a UV spectrophotometer (UV-2450, SHIMADZU, Kyoto, Japan) at 700 nm. The extracts of H_2_O-IP, NaHCO_3_-IP, NaOH-IP, and HCl-IP were combined in a 5:1 ratio and subjected to (NH_4_)_2_S_2_O_8_-H_2_SO_4_. The mixture was then autoclaved at 121 °C for 1 h. TP content of this form (H_2_O-P, NaHCO_3_-P, NaOH-P, HCl-P) was also obtained using a UV spectrophotometer (UV-2450, SHIMADZU, Kyoto, Japan) at 700 nm, with the result expressed as organic P (OP = TP−IP). The available P (AP) is obtained as Σ H_2_O-IP + H_2_O-OP + NaHCO_3_-IP + NaHCO_3_-OP; moderately available P (MAP) is equivalent to the result of Σ NaOH-IP + NaOH-OP; and non-available P (NAP) is Σ HCl-IP + HCl-OP + Residual-P [23].

### 2.5. Metagenomic Sequencing

DNA extraction and metagenomic shotgun sequencing were outsourced to Nanjing Personalbio Technology Co., Ltd. (Nanjing, China). Using the Qiagen DNeasy PowerSoil Kit (Omega Bio-tek, Norcross, GA, USA), 0.2000 g of genomic DNA was extracted from −80 °C frozen compost samples. DNA quality and quantity were assessed via NanoDrop ND-1000 and 1% agarose gel electrophoresis. The extracted DNA was fragmented to 400 bp using Covaris M220, and a metagenomic library was constructed using NEXTFLEX Rapid DNA-Seq (Illumina, San Diego, CA, USA). Sequencing was performed on the Illumina HiSeq platform (San Diego, CA, USA). Species annotations were obtained by aligning unigenes with the NR database, and abundance and differential analyses were performed using GO and KEGG databases. The sequences were submitted to the NCBI (PRJNA1189270).

### 2.6. Data Processing

Data were processed in Microsoft Excel 2003, and graphs were plotted using Origin 2022. Pearson correlation and one-way ANOVA analyses were conducted with SPSS 27 (Chicago, IL, USA). Species composition, Chao 1, Shannon, and Pielou’s evenness index, along with NMDS analysis, were performed on the Personalbio Gene Cloud. To investigate the relationship between phosphorus species and dominant bacterial phyla, we employed the “cor()” function in R software (Version 4.2.3) to conduct a Pearson correlation analysis.

## 3. Results and Discussion

### 3.1. Characterization of BC-Fe_3_O_4_NPs

**Figure 1:**
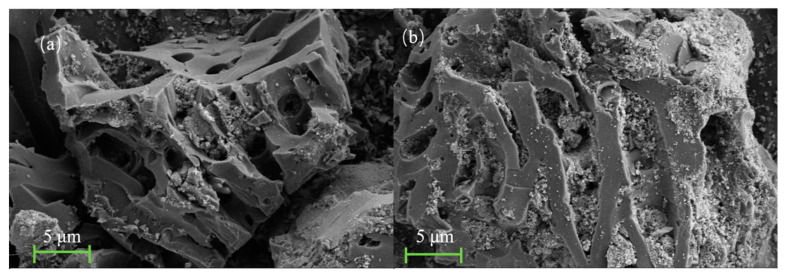
SEM images of BC (**a**) and BC-Fe_3_O_4_NPs (**b**).

**Figure 2:**
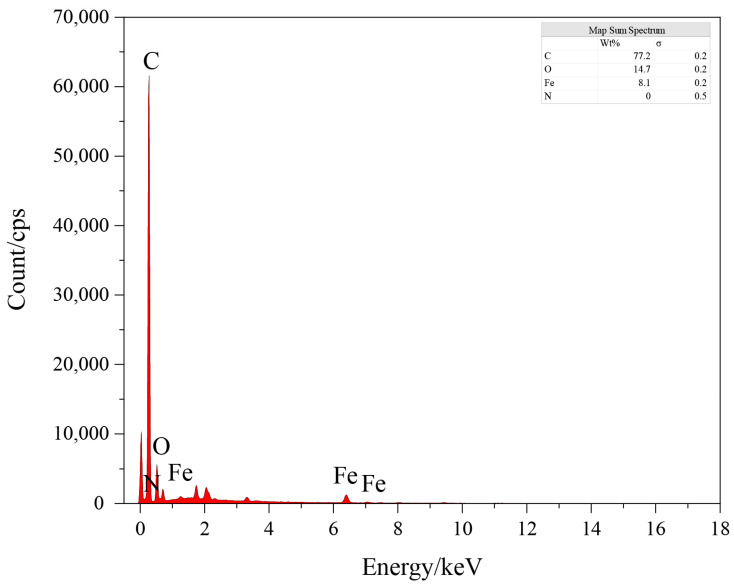
Mapping of BC-Fe_3_O_4_NPs.

Figure 1 presents the SEM images of BC (Figure 1a) and BC-Fe_3_O_4_NPs (Figure 1b), respectively. As observed in Figure 1, the surface of BC is relatively smooth and exhibits a porous structure, which provides abundant adsorption sites for nanomaterials [24]. After the loading experiment, spherical particles can be seen within the pores of BC, suggesting the successful loading of Fe_3_O_4_NPs onto BC. The EDS spectrum (Figure 2) reveals that the BC-Fe_3_O_4_NPs material is rich in C, O, and Fe elements, accounting for 77.2%, 14.7%, and 8.1%, respectively, confirming the successful loading of Fe_3_O_4_NPs.

According to the BET data presented in Table 1, the specific surface area of BC-Fe_3_O_4_NPs after successful loading (78.160 m^2^·g^−1^) is greater than that of BC (43.959 m^2^·g^−1^), and the average pore volume (0.194 cm^3^·g^−1^) is also larger than that of BC (0.069 cm^3^·g^−1^). The increase in particle number leads to an increase in the total particle surface area, resulting in an enhanced specific surface area of the composite material. The larger specific surface area and porous structure facilitate improved oxygen circulation during the aerobic composting process, thereby reducing the occurrence of anaerobic conditions during composting [25].

### 3.2. Changes in Physicochemical Properties During Composting

**Figure 3:**
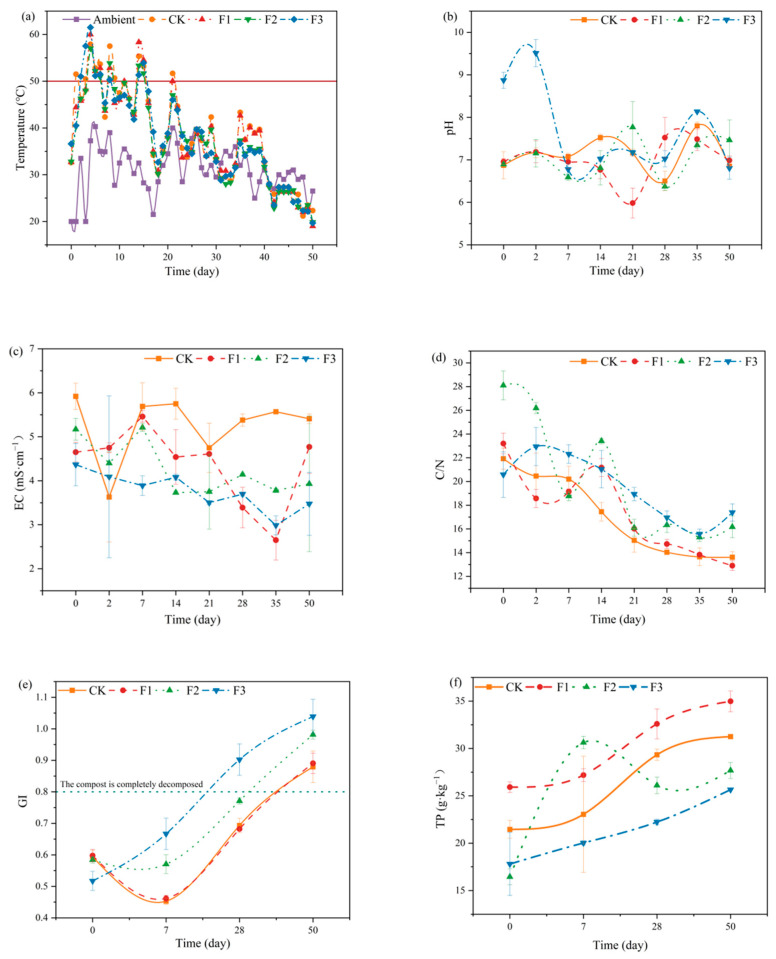
Changes in physical and chemical properties of compost in different periods. Note: (**a**) Temperature, (**b**) pH, (**c**) Electrical Conductivity (EC), (**d**) Carbon to Nitrogen Ratio (C/N), (**e**) Germination Index and (**f**) Total Phosphorus.CK: BC-Fe_3_O_4_NPs 0%; F1: BC-Fe_3_O_4_NPs 1%; F2: BC-Fe_3_O_4_NPs 5%; F3: BC-Fe_3_O_4_NPs 5% + CaO_2_ 5%.

As illustrated in Figure 3a, all treatments maintained temperatures above 50 °C for five days, ensuring the composting process met the requirements for harmlessness [26]. Specifically, the peak temperatures for the CK, F1, F2, and F3 treatments were 57.91 °C, 60.00 °C, 57.00 °C, and 61.50 °C, respectively. This indicates that the addition of BC-Fe_3_O_4_NPs can elevate the maximum composting temperature, although excessive amounts may inhibit this increase. The maximum temperature achieved in the F3 treatment is intricately linked to the addition of CaO_2_, possibly attributable to the decomposition of organic matter by CaO_2_, which subsequently provides essential nutrients for the growth and metabolic activities of microorganisms [27].

The initial pH values of the treatments ranged from 6.87 to 8.87(Figure 3b). During the thermophilic phase of composting (days 0–7), the pH of all treatments initially rose and then declined, likely due to the production of small organic acids through microbial metabolism [28]. In the high-temperature phase (days 7–14), except for the F1 treatment, the pH of CK, F2, and F3 increased, attributed to the rapid degradation of organic matter and the subsequent production of NH_3_ [24]. During the maturation phase (days 28–50), the pH of the compost first increased and then decreased, possibly due to the release and volatilization of amines following organic matter degradation, leading to the formation of organic and inorganic acids. At the end of composting, the pH ranged from 6.80 to 7.46, indicating a weakly alkaline environment, which complies with the Chinese standard for mature organic fertilizers (5.5 < pH < 8.5, NYT525-2021 [29]).

The electrical conductivity (EC) values of the treatments during composting generally followed a trend of initial decrease, subsequent increase, and final decrease (Figure 3c), consistent with the findings of Zhang [30]. The F1 and F3 treatments reached their lowest EC values (2.65 mS·cm^−1^) on day 35. This may be attributed to the 1% BC-Fe_3_O_4_NPs promoting microbial growth and metabolism, facilitating the conversion of organic matter to humus, while CaO_2_ combined with small organic acids, thereby reducing the EC value. At the end of composting, the EC values of all treatments ranged from 3.48 to 5.41 mS·cm^−1^, meeting the compost maturity standard (EC < 9.00 mS·cm^−1^) [31].

Carbon and nitrogen sources serve as energy and nutrient sources for microorganisms, and the C/N ratio is used to evaluate compost maturity [11,32]. The C/N ratio of all treatments decreased over time (Figure 3d). At the end of composting, the C/N ratios for CK, F1, F2, and F3 were 13.61, 12.90, 16.17, and 17.39, respectively, all below the threshold of 20, indicating maturity [12]. Notably, compared to CK, F2 and F3 had higher C/N ratios, while F1 had a lower C/N ratio. This suggests that excessive addition of carbon-based materials may inhibit microbial growth and metabolism, leading to an increase in the C/N ratio of the compost.

As an essential indicator for assessing compost maturity, the *GI* serves to determine the presence of phytotoxicity in compost. It is generally accepted that compost is considered basically mature and non-phytotoxic when *GI* exceeds 50% and fully mature when *GI* surpasses 80% [33,34]. The variation of *GI* during the composting process for different treatments is illustrated in Figure 3e. On day 0, the *GI* values for the treatments were 59.38%, 59.77%, 58.39%, and 51.72%, respectively. As composting progressed, a decline in *GI* to the lowest points of 45%, 46%, and 57% was observed for CK, F1, and F2 treatments, possibly due to the extensive decomposition of organic matter into toxic intermediates, such as NH_3_ and organic acids [35,36]. By the end of the composting period, the *GI* values for all treatments had reached 87.93%, 89.06%, 164%, and 104%, respectively, fulfilling the criteria for complete compost maturity. Notably, the *GI* of the F3 treatment progressively increased over time, achieving full maturity requirements by day 28, which may be attributed to the elevated temperatures induced by CaO_2_ addition. These results suggest that the incorporation of BC-Fe_3_O_4_NPs accelerates the aerobic fermentation process, and, in particular, the combined addition of BC-Fe_3_O_4_NPs and CaO_2_ enhances the detoxification of compost materials, leading to superior-quality fermentation products.

The TP content across different treatments during the composting process is illustrated in Figure 3f. At the conclusion of the composting period, the TP contents for each treatment were 31.25 g·kg^−1^, 34.98 g·kg^−1^, 27.68 g·kg^−1^, and 25.65 g·kg^−1^, respectively. This increase in TP content can be attributed to the concentration effect that occurs during composting [21]. Notably, the TP content in the F3 treatment was the lowest at the end of composting. This could be due to the adsorption–complexation of phosphorus through the addition of 5% BC-Fe_3_O_4_NPs and 5% CaO_2_, which resulted in a relatively lower TP content in the F3 treatment compared to the other treatments.

### 3.3. Transformation of P Forms

**Figure 4:**
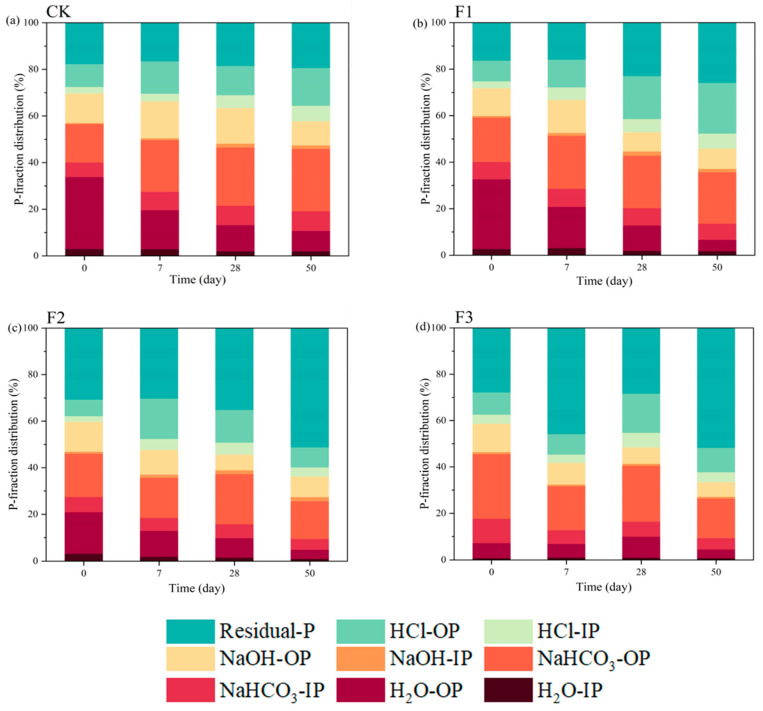
Changes of phosphorus components. Note: (**a**) CK: BC-Fe_3_O_4_NPs 0%; (**b**) F1: BC-Fe_3_O_4_NPs 1%; (**c**) F2: BC-Fe_3_O_4_NPs 5%;(**d**) F3: BC-Fe_3_O_4_NPs 5% + CaO_2_ 5%.

The sequential extraction of P using different extracts reveals the dynamics of various P fractions during composting (Figure 4) [37]. In the CK, the P forms undergoing significant changes were H_2_O-OP and NaHCO_3_-OP. As composting progressed, the proportion of H_2_O-OP decreased, while that of NaHCO_3_-OP increased. By the end of composting, the predominant P forms in the CK treatment were NaHCO_3_-OP, Residual-P, and HCl-OP, accounting for 26.76%, 16.23%, and 19.32%, respectively. Notably, NaHCO_3_-OP is easily absorbed by plants, Residual-P is the most stable form, and HCl-OP represents Ca-bound P [38]. The P forms in the F1 treatment at the end of composting were similar to those in the CK treatment, dominated by Residual-P, NaHCO_3_-OP, and HCl-OP, with proportions of 25.81%, 21.83%, and 22.14%, respectively. This suggests that the addition of BC-Fe_3_O_4_NPs promoted the transformation of P towards more stable forms. In the F2 treatment, at the end of the high-temperature phase of composting (day 28), the primary P forms were Residual-P, NaHCO_3_-OP, and HCl-OP, accounting for 35.10%, 21.46%, and 14.05%, respectively. By the end of composting, the proportion of Residual-P increased to 51.16%, NaHCO_3_-OP increased to 16.15%, and HCl-OP decreased to 8.64%. This indicates that the addition of 5% BC-Fe_3_O_4_NPs significantly increased the proportion of Residual-P among the P forms. The trend in P form changes in the F3 treatment was similar to that in the F2 treatment. At the initial stage of composting in the F3 treatment, Residual-P, NaHCO_3_-OP, and HCl-OP accounted for 27.74%, 27.81%, and 12.21%, respectively. During the heating phase, the proportion of Residual-P increased to 45.76%, while the proportions of other forms decreased. This suggests that during the heating phase, P forms became more stable, possibly due to the increased pH resulting from the addition of CaO_2_, which enhanced phosphate adsorption [39]. In the high-temperature phase, the proportion of Residual-P decreased to 28.28%, while the proportions of H_2_O-OP, NaHCO_3_-OP, and HCl-OP increased. This may be attributed to the gradual conversion of CaO_2_ into other substances, such as Ca(OH)_2_, leading to oxygen release, which stimulated the activity of certain microorganisms, activating P and converting it into more labile forms. By the end of composting, the proportion of Residual-P increased to 51.68%, while the proportions of other forms decreased. This could be because during the cooling phase, with the reduction in moisture content of the compost pile, excess metal cations could not form their oxides or hydroxides and instead combined with free phosphate ions to form insoluble P precipitates [40], driving the transformation of P towards more stable forms.

### 3.4. Changes in Bacterial Community Structure During the Composting Process

**Figure 5:**
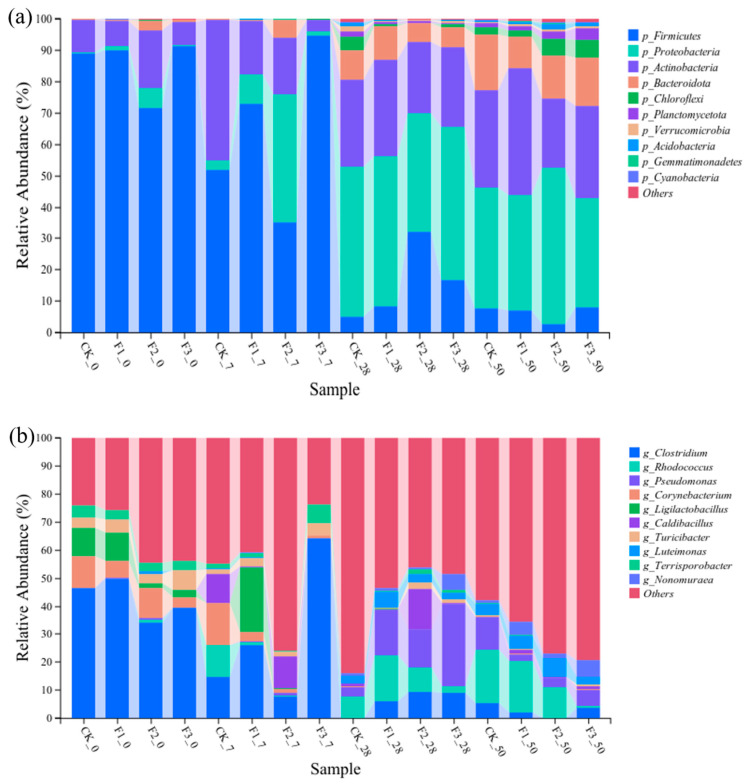
Changes of bacterial communities at phyla (**a**) and genus (**b**) levels during composting. Note: CK: BC-Fe_3_O_4_NPs 0%; F1: BC-Fe_3_O_4_NPs 1%; F2: BC-Fe_3_O_4_NPs 5%; F3: BC-Fe_3_O_4_NPs 5% + CaO_2_ 5%. 0, 7, 28, and 50 represent the composting time points.

Figure 5a illustrates the distribution of bacterial communities at the phylum level across various treatments. Throughout the composting process, *Firmicutes*, *Actinobacteria*, and *Proteobacteria* emerged as the dominant phyla, albeit with varying relative abundances at different stages [41]. On day 0 of composting, *Firmicutes* predominated in all treatments, accounting for 88.93%, 90.11%, 71.56%, and 91.15%, respectively.

As composting progressed to day 7, the relative abundances of *Actinobacteria* and *Proteobacteria* increased across treatments, leading to differences in bacterial community structures. Among all treatments, *Firmicutes* remained the dominant phylum in F1 (72.89%) and F3 (94.81%), while *Proteobacteria* prevailed in F2, accounting for 41.01%. This variation can be attributed to the thermotolerance of *Firmicutes*, which enables them to withstand extreme high temperatures [42]. The differing maximum temperatures achieved in each treatment resulted in varied proportions of *Firmicutes* during this period. By day 28 of composting, the bacterial communities in all treatments shifted, with *Proteobacteria* replacing *Firmicutes* and *Actinobacteria* as the dominant phylum. By day 50, the bacterial communities in all treatments stabilized, characterized by an increase in the relative abundance of *Bacteroidetes*, a continued decrease in *Firmicutes*, and stable levels of *Actinobacteria* and *Proteobacteria*.

As shown in Figure 5b, at the genus level, during the initial stage of composting, the dominant genera were primarily *Clostridium*, *Corynebacterium*, and *Ligilactobacillus*. By day 7, the relative abundance of *Clostridium* decreased, resulting in differences in dominant genera across treatments. In the CK treatment, *Corynebacterium* and *Clostridium* were the dominant genera, accounting for 14.91% and 14.70%, respectively; in F1, *Ligilactobacillus* and *Clostridium* predominated, with proportions of 23.13% and 26.10%, respectively; F2 was dominated by *Caldibacillus*, accounting for 11.40%; and, in F3, *Clostridium* remained the dominant genus, accounting for 64.13%. *Corynebacterium* belongs to *Actinobacteria*, while *Clostridium* and *Ligilactobacillus* belong to *Firmicutes*. The former possesses the ability to decompose organic matter, while the latter can produce spores to resist desiccation and extreme environments. During the cooling and maturation phases of composting, *Rhodococcus* emerged as the dominant genus across all treatments. This genus, commonly found in the later stages of composting, has been closely associated with compost maturity [43].

### 3.5. Analysis of Bacterial Community Richness and Diversity During the Composting Process

As shown in Table 2, the Coverage index exceeded 0.999 for all samples across different composting stages, indicating that the sequencing results accurately represent the true composition of the samples [44]. The Chao 1 index reflects the richness of bacterial communities in the samples. On day 0, the Chao 1 indexes for treatments F1, F2, and F3 were all higher than that of the CK treatment. As composting progressed to day 28, the CK treatment exhibited a higher Chao 1 index compared to the other treatments. This could be attributed to the higher maximum temperatures and prolonged periods of high temperatures in treatments F1, F2, and F3 compared to CK. During the high-temperature phase, environmental stress significantly intensifies, allowing thermophilic microorganisms to dominate the ecology due to their unique heat resistance, while other non-thermophilic microorganisms die off in large numbers due to their inability to adapt to extreme high temperatures, resulting in a significant decrease in microbial community richness [45]. By day 50, the Chao 1 indexes for treatments F1, F2, and F3 were 35,953.68, 35,621.30, and 36,460.46, respectively, all exceeding that of the CK treatment (34,696.03). According to the Tukey’s HSD test, the addition of BC-Fe_3_O_4_NPs and CaO_2_ increased the richness of bacterial communities, with the best effect observed for the addition of 1% BC-Fe_3_O_4_NPs.

The Shannon index primarily reflects the diversity of the sample community, influenced by both species’ richness and evenness within the sample community [46]. The Shannon index followed a similar pattern as the Chao 1 index across treatments. On day 0, the Shannon indexes for treatments F1, F2, and F3 were all higher than that of the CK treatment. By day 28, the CK treatment exhibited a higher Shannon index compared to the other treatments. At the end of composting, the Shannon index for treatment F3 was 6.65, which was higher than that of the other treatments. This is consistent with the findings of Tian, who reported that the microbial diversity in matured compost samples is the most abundant.

### 3.6. NMDS Analysis of Bacterial Communities During the Composting Process

**Figure 6:**
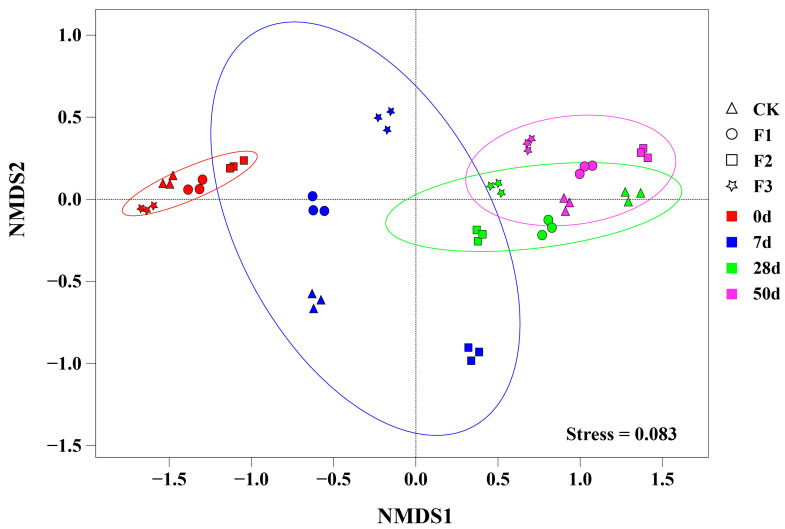
NMDS analysis of metagenomic sequencing data for each process. Note: CK: BC-Fe_3_O_4_NPs 0%; F1: BC-Fe_3_O_4_NPs 1%; F2: BC-Fe_3_O_4_NPs 5%; F3: BC-Fe_3_O_4_NPs 5% + CaO_2_ 5%. 0, 7, 28, and 50 represent the composting time points.

NMDS (Non-Metric Multidimensional Scaling) analysis is an advanced data analysis technique that skillfully projects complex multidimensional data into a simplified low-dimensional space for precise positioning, in-depth analysis, and reasonable classification, while ensuring that the original relationships between the research objects are fully preserved [47]. As shown in Figure 6, during the initial stage of composting, the bacterial communities of various treatments exhibited a closely clustered state. This phenomenon may be attributed to the additives not yet fully interacting with the main compost material. As composting progressed and the temperature of the compost pile gradually increased, by day 7, significant differences in bacterial communities were observed among the samples from different treatments. This change clearly indicated the profound impact of BC-Fe_3_O_4_NPs on the bacterial community structure within the composting system [48].

Further observation throughout the entire composting cycle revealed that apart from the subtle differences in bacterial communities among treatments during the initial stage, it is noteworthy that both the F2 and F3 treatments showed a trend of being further away from CK on the NMDS plot compared to the F1 treatment. This finding strongly suggests that the addition schemes of 5% BC-Fe_3_O_4_NPs and 5% BC-Fe_3_O_4_NPs + 5% CaO_2_ had a more profound and lasting impact on the bacterial communities during composting compared to the addition of 1% BC-Fe_3_O_4_NPs.

### 3.7. Correlation Between Phosphorus Forms and Microbial Communities During the Composting Process

**Figure 7:**
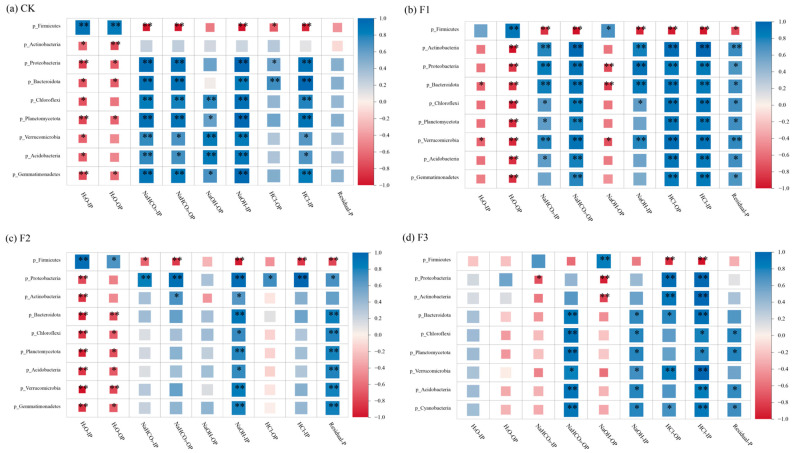
Correlation between the top 10 relative abundances of bacterial phyla and phosphorus speciation at the phylum level. Note: CK: BC-Fe_3_O_4_NPs 0%; F1: BC-Fe_3_O_4_NPs 1%; F2: BC-Fe_3_O_4_NPs 5%; F3: BC-Fe_3_O_4_NPs 5% + CaO_2_ 5%. IP and OP denote the inorganic and organic forms of phosphorus, respectively. H_2_O-IP and H_2_O-OP represent the phosphorus fractions extracted using H_2_O, while NaHCO_3_-IP and NaHCO_3_-OP signify the phosphorus fractions extracted with NaHCO_3_. Similarly, NaOH-IP and NaOH-OP indicate the phosphorus forms obtained through NaOH extraction, and HCl-IP and HCl-OP represent those extracted using HCl. Lastly, Residual-P refers to the phosphorus remaining in its residual form. * *p* < 0.05, ** *p* < 0.01.

During the aerobic composting of pig manure, the transformation of phosphorus forms is not only profoundly influenced by the physicochemical characteristics of the compost but also closely related to the growth and metabolic activities of microorganisms. A deep exploration of the interaction between phosphorus forms and microbial communities is crucial for unveiling the phosphorus transformation mechanisms within the composting system. In this study, Pearson correlation analysis was employed, with the results presented in Figure 7, to delve into the correlations between phosphorus forms and dominant bacterial phyla under different treatment conditions.

In the CK treatment, *Firmicutes* showed a significant positive correlation with H_2_O-IP and H_2_O-OP (*p* < 0.01) and a negative correlation with other phosphorus forms, although the correlations with NaOH-OP and Residual-P were not significant. Other dominant bacterial phyla exhibited opposite relationships with phosphorus forms compared to *Firmicutes*, showing negative correlations with H_2_O-IP and H_2_O-OP and positive correlations with other forms. It is noteworthy that the correlations between various dominant bacterial phyla and Residual-P were not significant (*p* > 0.05), and *Actinobacteria* only showed a significant negative correlation with H_2_O-IP and H_2_O-OP (*p* < 0.01).

*Actinobacteria* play a crucial role in organic matter decomposition, and the addition of BC-Fe_3_O_4_NPs enhanced the significant correlations between *Actinobacteria* and other phosphorus forms. However, as the addition amount increased, it altered the correlations between the bacterial community and different phosphorus forms. In the F1 treatment, the correlations between various bacterial phyla and phosphorus forms were more significant. This may be attributed to the reduction of Fe^3+^ to Fe^2+^, which intensified the composting fermentation process, increasing substrate conversion and bacterial growth [49]. Unlike the CK treatment, in the F1 treatment, *Firmicutes* showed a significant positive correlation with NaOH-OP (*p* < 0.05), while other dominant bacterial phyla exhibited negative correlations with NaOH-OP. Studies have shown that *Firmicutes* are widely distributed during composting and have a significant impact on TOC degradation, compost humification [50], and the availability and mobility of phosphorus in the system. When *Firmicutes* dominate during the high-temperature stage, the phosphate released from TOC degradation may be adsorbed and fixed by BC-Fe_3_O_4_NPs [51], forming NaOH-P.

In the F2 treatment, the correlations between dominant bacterial phyla and phosphorus forms further changed. Specifically, *Proteobacteria* and *Bacteroidota* showed a positive correlation with HCl-OP, while other bacterial phyla exhibited a negative correlation with this phosphorus form (*p* > 0.05). When CaO_2_ was added as a co-agent, the dominant bacterial phyla showed negative correlations with H_2_O-OP, NaHCO_3_-IP, and NaOH-OP (*p* > 0.05). This may be due to the favorable conditions provided by CaO_2_ for phosphate adsorption, promoting the transformation from NaOH-OP to HCl-OP [52].

## 4. Conclusions

In summary, the addition of BC-Fe_3_O_4_NPs, especially in combination with CaO_2_, significantly influenced the composting process by increasing peak temperatures, promoting the transformation of phosphorus into more stable forms, altering the bacterial community structure, and enhancing bacterial diversity and richness. Furthermore, the correlations between the bacterial community and phosphorus forms were modulated by the addition of BC-Fe_3_O_4_NPs, with different effects observed at different addition levels and in combination with CaO_2_. These findings provide valuable insights for optimizing composting processes and improving phosphorus utilization efficiency in agricultural practices.

## Figures and Tables

**Table 1:** BET analysis of materials.

Samples	Specific Surface Area (m^2^·g^−1^)	Pore Volume (cm^3^·g^−1^)
BC-Fe_3_O_4_NPs	78.160	0.194
BC	43.959	0.069

**Table 2:** Abundance and diversity of bacterial communities during composting.

Day/Treatment		Chao 1 Index	Shannon Index	Coverage Index
0 d	CK	19,670.29 ± 213.62 a	3.82 ± 0.00 a	0.9995 ± 0.0000 a
	F1	27,922.72 ± 121.10 b	4.17 ± 0.00 b	0.9993 ± 0.0000 b
	F2	32,918.24 ± 59.39 c	5.08 ± 0.02 c	0.9994 ± 0.0000 c
	F3	25,831.02 ± 310.25 d	4.53 ± 0.01 d	0.9993 ± 0.0000 d
7 d	CK	29,042.54 ± 160.90 a	4.86 ± 0.01 a	0.9993 ± 0.0000 a
	F1	31,039.06 ± 256.56 b	4.61 ± 0.00 b	0.9994 ± 0.0000 b
	F2	29,907.30 ± 250.14 c	4.81 ± 0.01 a	0.9994 ± 0.0000 c
	F3	24,591.30 ± 290.04 d	3.64 ± 0.00 c	0.9993 ± 0.0000 a
28 d	CK	36,468.00 ± 448.36 a	6.53 ± 0.00 a	0.9996 ± 0.0000 a
	F1	34,037.88 ± 133.84 b	5.73 ± 0.00 b	0.9995 ± 0.0000 ab
	F2	33,430.68 ± 254.52 b	5.54 ± 0.01 c	0.9995 ± 0.0000 b
	F3	34,000.10 ± 242.25 b	5.64 ± 0.00 d	0.9996 ± 0.0000 ab
50 d	CK	34,696.03 ± 193.89 a	6.15 ± 0.01 a	0.9996 ± 0.0000 a
	F1	35,953.68 ± 116.20 b	6.36 ± 0.00 b	0.9996 ± 0.0000 a
	F2	35,621.30 ± 129.33 b	5.92 ± 0.00 c	0.9996 ± 0.0000 a
	F3	36,460.46 ± 81.74 c	6.65 ± 0.00 d	0.9993 ± 0.0000 a

Note: Different lowercase letters indicate significant differences according to Tukey’s HSD test (*p* < 0.05). CK: BC-Fe_3_O_4_NPs 0%; F1: BC-Fe_3_O_4_NPs 1%; F2: BC-Fe_3_O_4_NPs 5%; F3: BC-Fe_3_O_4_NPs 5% + CaO_2_ 5%.

## Data Availability

All data are included in the article.

## Supplementary Materials

The following supporting information can be downloaded at https://www.mdpi.com/article/10.3390/microorganisms12122593/s1, Table S1. Physical and chemical properties of test materials. Figure S1. Schematic Diagram of Aerobic Composting Device.
