# Supplementary material for: Exploring the Influence of Biochar-Supported Nano-Iron Oxide on Phosphorus Speciation Transformation and Bacterial Community Structure in Aerobic Pig Manure Composting Processes
Source: Microorganisms. 2024 Dec 14;12(12):2593. doi: 10.3390/microorganisms12122593 (PMC11677891; doi:10.3390/microorganisms12122593)
Supplement: Supplementary file 1 [file microorganisms-12-02593-s001.zip › microorganisms-3335012-supplementary.pdf]

Table S1. Physical and chemical properties of test materials.

Materials	pH	EC (ms·cm^{-1})	Moisture content (%)	TC (%)	TN (%)	C/N
Pig manure	6.16±0.02	5.85±0.27	80.23±0.02	39.38±0.52	3.96±0.01	43.07±1.80
Maize straw	5.74±0.01	2.47±0.05	10.07±0.05	39.86±0.05	0.93±0.04	9.95±0.12
BC-Fe_3O_4NPs	6.80±0.01	1.20±0.02	–	37.23±0.33	0.54±0.01	68.40±0.03

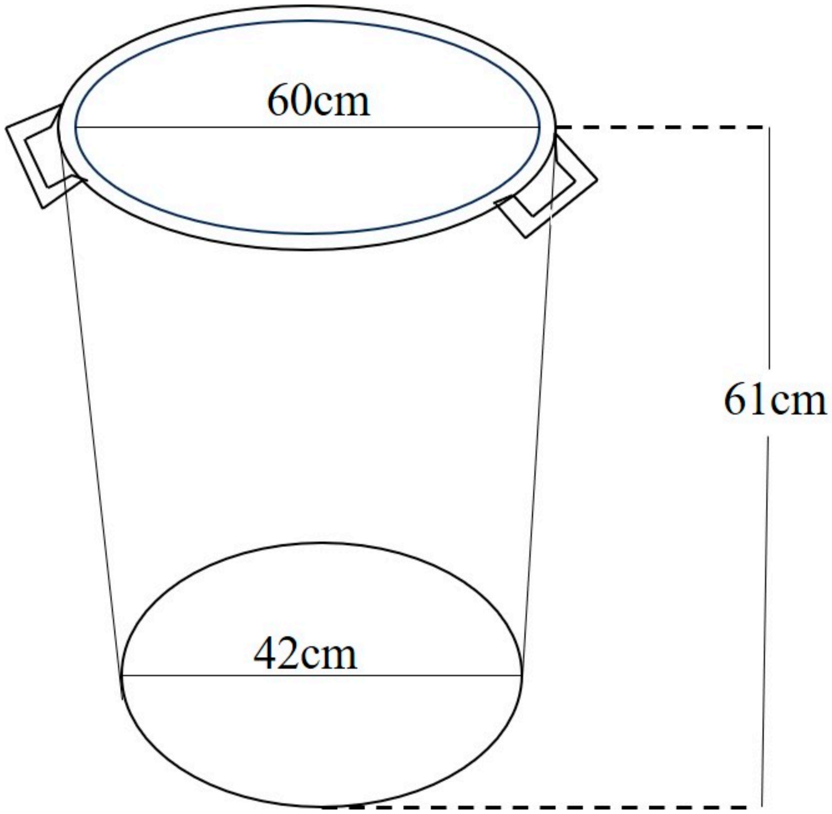

Figure S1. Schematic Diagram of Aerobic Composting Device.
